# Separation of Organic Carbon and Nutrients from Liquid Waste by Using Membrane Technologies

**DOI:** 10.3390/membranes16020071

**Published:** 2026-02-20

**Authors:** Stanislas Ndayishimiye, Samuel Bunani, Emery Nkurunziza, Nalan Kabay

**Affiliations:** 1 CRSNE—Research Center in Natural Sciences and Environment, Faculty of Sciences, University of Burundi, Bujumbura P.O. Box 2700, Burundi; stanislasndayishimiye2@gmail.com (S.N.); emery.nkurunziza@ub.edu.bi (E.N.); 2 Chemical Engineering Department, Ege University, Izmir 35040, Turkey; nalan.kabay@ege.edu.tr

**Keywords:** microfiltration, ultrafiltration, organic carbon fractionation, nutrient speciation, wastewater resource recovery, membrane mechanisms

## Abstract

Rising concentrations of organic carbon (OC), phosphorus, and nitrogen in liquid waste from urban, industrial, and agricultural sources pose persistent challenges for environmental protection and resource recovery. Despite extensive application of microfiltration (MF) and ultrafiltration (UF) in wastewater treatment, their role in selective organic carbon and nutrient fractionation remains insufficiently clear-cut and is often interpreted solely through nominal pore size. This review was guided by the hypothesis that the reported limitations of MF and UF for nutrient separation are not intrinsic to the technologies but arise from simplified interpretations of separation mechanisms. A unified analytical framework was developed by synthesizing recent studies, linking membrane surface charge, pore structure, solute speciation, fouling-induced secondary layers, and operating conditions to the observed separation behavior. The analysis shows that MF fractionates particulate OC and suspended solids, whereas UF extends separation to macromolecular OC and phosphorus mainly via indirect retention mechanisms. Dissolved nitrogen species largely permeate both membranes unless they are transformed into retainable forms. Performance differences between MF and UF are conditional and system-dependent, with enhanced selectivity emerging through process integration. MF and UF can thus be repositioned as strategic fractionation interfaces within integrated treatment systems supporting circular economy–oriented wastewater management.

## 1. Introduction

The growing global demand for water and the increasing generation of wastewater from domestic, industrial, and agricultural sources have intensified the need for efficient and sustainable treatment technologies [1,2,3].

Liquid waste streams often contain significant concentrations of OC and nutrients such as nitrogen and phosphorus, which, if not properly managed, contribute to serious environmental issues including eutrophication, groundwater contamination, and greenhouse gas emissions [4,5]. Conversely, these waste streams also represent a valuable source of recoverable materials that could be reused in agriculture and industry, supporting the principles of circular economy and resource recovery [6,7,8]. Conventional wastewater treatment methods, including biological and chemical processes, have proven effective in many cases but often present limitations such as high energy consumption, sludge generation, and incomplete removal of certain pollutants [9]. Among advanced treatment options, pressure-driven membrane technologies, particularly MF and UF, have gained attention due to their modularity, operational efficiency, and ability to selectively separate particulate and colloidal matter through size exclusion [10,11].

MF and UF membranes differ in pore size and separation performance, with MF typically removing particles > 0.1 micrometer (µm) and UF targeting smaller solutes, including macromolecules and certain nutrient forms [12,13]. These membranes can effectively retain OC compounds and, to a certain extent, phosphorus and nitrogen species, depending on membrane material, configuration, and operational parameters [14,15,16]. Additionally, integrating MF and UF with pre- or post-treatment techniques such as coagulation, adsorption, or biological processes enhances the removal efficiency of nutrients, making these technologies suitable for both centralized and decentralized treatment systems [11,17]. This review critically examines MF and UF membranes through a hypothesis-driven framework, focusing on how membrane properties, feed composition, and operating conditions govern selective separation. The objective is to highlight the advantages and limitations of these technologies and inform future researchers the strategies for sustainable wastewater treatment and resource recovery.

This review tests the hypothesis that the apparent limitations of MF and UF in nutrient separation arise not from intrinsic membrane constraints, but from how separation mechanisms, solute speciation, and process integration are interpreted and applied.

## 2. Technologies for the Removal of OC and Nutrients from Wastewater

**Figure 1:**
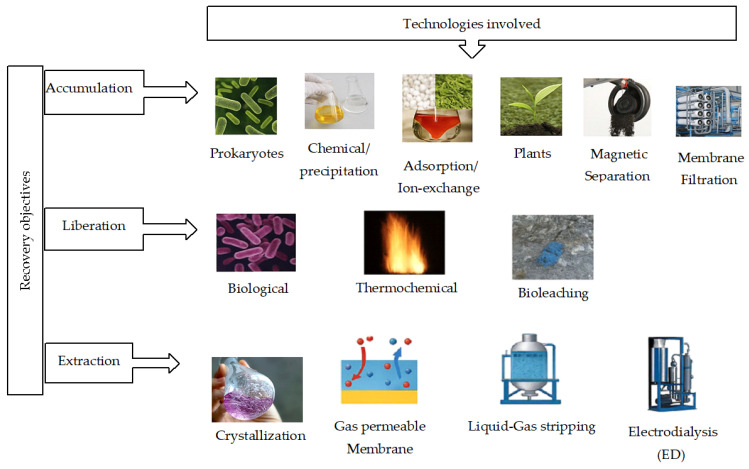
Technologies to remove nutrients from waste streams [20].

The elimination of OC and nutrients from wastewater is essential to obtain certain benefits, including preventing eutrophication of surface water bodies, recovering fertilizer, and maintaining water quality [18]. According to Deemter et al. (2022) [19], various technologies are employed to remove key nutrients like carbon, nitrogen, and phosphorus from wastewater and the recovery rate varies from one technology to another (Figure 1).

**Figure 2:**
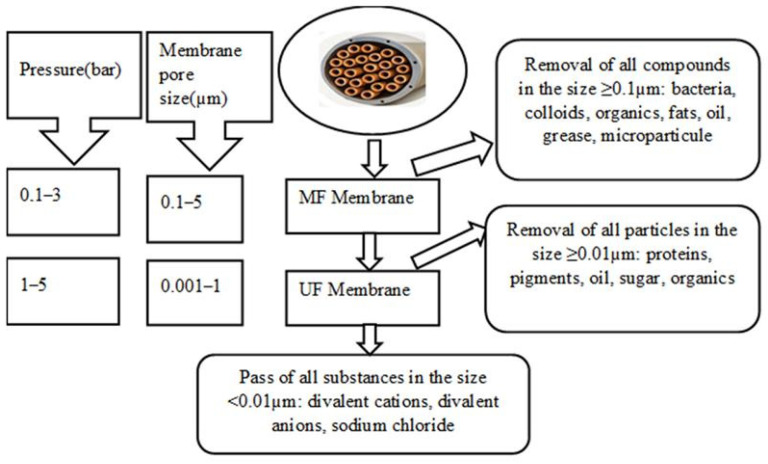
Physical and chemical features of the membranes considered.

Membrane filtration is widely applied when nutrient concentration or recovery is targeted [20]. Product recovery quality depends on membrane pore size, nutrient size, feed characteristics, and the pressure applied (Figure 2). MF and UF processes are used especially for the removal of organic substances from liquid waste [19,21,22]. In general, MF membranes are used to remove particles larger than 0.5 μm, whereas membrane filters with a pore size of 0.002–0.5 μm are available for UF in order to eliminate macromolecules and colloidal particles. According to Utoro et al. (2019) [23], both membranes are also applied for filtration of viruses (0.03–1 μm) and bacteria (0.5–20 μm). MF membranes have pore sizes ranging from 0.1 to 10 μm with an applied pressure range of 0.1–2 bar from an inlet fluid stream. Globally, MF can effectively remove suspended solids (SS) and particulate and colloidal organic species. However, it is less effective in removing dissolved organics, nutrients, and smaller organic compounds. UF membranes have finer pore structures than MF membranes, allowing them to retain smaller particles and dissolved macromolecules [24]. Due to the size exclusion mechanism, they can retain much smaller particles, including macromolecules and some colloidal substances. UF is effective in removing a broader range of organic contaminants, including proteins, polysaccharides, and other macromolecules [25,26]. It can also remove some dissolved organic compounds, depending on their size [27]. For a high efficiency to retain inorganic and organic micropollutants, MF and UF are applied in integrated systems coupled with coagulation, flocculation, sedimentation, adsorption, complexion with polymers or surfactants, and biological reactions [28,29]. With low operating pressure in the range of 0.1–2.5 bar and pore size ranging between 0.1 and 10 μm, MF membranes removed organic compounds up to 95% by showing a water permeability of 500 L/m^2^·h·bar [28]. On the other hand, with UF membranes, the same recovery was achieved by applying a pressure of 2–5 bar with 0.001–1 µm of membrane pore size by showing 150 L/m^2^·h·bar of permeability [28,30]. A high concentration of organics such as OC is found in the concentrate side of MF when municipal, urban, and agricultural wastewater are treated and inorganic ions remain in the MF permeate [31,32]. Phosphorous and nitrogen in particulate form with size > 0.1 μm can be selectively removed by those filtration processes [33].

In their research, Refs. [31,34,35] demonstrated that organics are retained by microfiltration and ultrafiltration membranes while inorganic ions pass through the pore of each module. When MF is involved, OC is retained whereas inorganics such as phosphorus and nitrogen forms pass through the membrane pore size [35,36]. Inorganic separation or recovery in the UF membranes depends on the ionic charge [37]. Total nitrogen (TN) and total phosphorus (TP) dominated by particulate forms show a high concentration in the UF concentrate [38]. However, monovalent and divalent ions for instance are found in the UF permeate [39]. This behavior depends on the size of the components (Table 1) and the membrane solute permeability.

This table demonstrates that the reported separation performances cannot be interpreted solely on the basis of nominal pore size or membrane classification (MF and UF). Variations in membrane material, surface charge, and configuration introduce secondary separation mechanisms that influence organic carbon and nutrient retention. Hydrophilic surfaces and asymmetric structures tend to enhance organic matter interaction and fouling layer development, indirectly improving nutrient retention. Consequently, membrane characteristics define not only hydraulic performance but also the physicochemical environment governing selective separation, explaining part of the variability observed across studies.

## 3. Removal Efficiency of OC Compounds

### 3.1. Evaluating OC Removal Efficiency in Liquid Waste via MF Membranes

In the context of assessing the removal efficiency of OC from liquid waste, MF emerges as a promising option, offering distinctive operational advantages and limitations [42,43]. By harnessing a combination of physical sieving and adsorption mechanisms, this approach has demonstrated effectiveness in removing organic carbon from liquid waste [44]. The process mainly depends on the pore size, which is usually between 0.1 and 10 μm. This helps to physically separate particulate organic matter from the liquid phase [45]. The liquid waste passes through the membrane, and particles exceeding the effective pore size are retained via surface sieving and internal pore blocking mechanisms [46]. This makes the water clearer and removes a lot of the suspended organic carbon [47]. As shown in Table 2, the efficiency of microfiltration in removing organic carbon depends on several factors, such as the feed water content and how the system is operated [47,48]. The membrane material also significantly impacts the removal efficiency [49]. Materials such as polyvinylidene fluoride (PVDF), polypropylene (PP), and ceramics offer different levels of hydrophobicity, chemical resistance, and mechanical strength [50,51]. These properties influence not only how well the membrane can filter out organic carbon but also its longevity and maintenance requirements [52]. In general, microfiltration represents a robust methodology for the removal of particulate and colloidal organic carbon [53,54]. This is achieved through the dual mechanisms of sieving and adsorption [25,55].The presence of organic carbon in liquid waste can be detected in a number of ways, including the use of chemical oxygen demand (COD), biological oxygen demand (BOD), total organic carbon (TOC), and dissolved organic carbon (DOC) [54]. MF membranes generally operated at transmembrane pressure (TMP) below 0.3 bar achieved a performance of 65–75% in term of TOC rejection [55].

### 3.2. Removal Efficiency of OC from Liquid Waste by Using UF Membranes

In the implementation of UF membranes for the removal of OC from liquid waste, their fine pore structure enables the retention of macromolecules and colloidal organic matter [13,64,65]. As water permeates through the porous structure of the membrane, larger organic molecules such as humic substances, proteins, and colloids are retained on the surface or in the pores [52]. Materials like polyethersulfone (PES), polysulfone (PS), cellulose acetate (CA), and various types of modified polymer blends are commonly used due to their favorable mechanical strength, chemical resistance, and ability to form consistent pore structures [66]. In exploring the removal efficiency of OC from liquid waste using UF membranes, several case studies and practical applications underscore the versatility and efficiency of this technology [67]. By integrating UF membranes into their treatment processes, many municipalities have achieved substantial reductions of OC levels, thereby enhancing the overall quality of discharged effluent [68]. Additionally, pilot projects in agricultural settings demonstrated its potential to manage runoff containing pesticides and fertilizers [69,70,71,72]. These projects have shown promising results in reducing organic load before water is released back into natural waterways or reused for irrigation purposes [73,74].

### 3.3. Comparative Behavior of MF and UF Toward Particulate and Macromolecular Organic Carbon

The results summarized in Table 2 and Table 3 indicate that both MF and UF achieve consistently high removal of organic carbon when OC is predominantly present in particulate or macromolecular forms. UF generally exhibits higher and more stable OC retention due to combined size exclusion and adsorption mechanisms. However, the marginal performance gap between MF and UF narrows in systems dominated by particulate organic carbon, suggesting that membrane selection should be driven by OC fractionation rather than total OC concentration. This highlights the importance of aligning membrane choice with organic carbon characteristics instead of assuming the inherent superiority of tighter membranes.

## 4. Removal Efficiency of Phosphorus from Liquid Waste

### 4.1. Microfiltration Performance Toward Different Phosphorus Forms

Conventional treatment methods often struggle to meet stringent discharge limits for nutrients, particularly phosphorus [85,86]. For effective removal of phosphorus compounds from liquid waste, MF serves as an effective pre-concentration step for particulate and colloidal phosphorus [87,88]. Combined with other methods such as biological treatment or chemical precipitation, it serves as an excellent pre-treatment step [89,90]. The MF membranes facilitate the concentration of phosphorus compounds by filtering out larger solids and colloidal particles that might otherwise interfere with subsequent treatment stages [89]. The practical implementation of MF membranes for the removal of phosphorus compounds from liquid waste has seen considerably success across various wastewater types (Table 4). By using a series of membrane modules with pore sizes optimized for capturing fine particulate matter and colloidal phosphorus, the facility achieved a reduction of total phosphorus levels to below 0.1 mg/L [90,91,92]. In addition, when combined with coagulation or adsorption, the process performance could be increased to 80–95% [93,94]. PO_4_^3−^ removal rates reached up to 11% for MF alone, 91% for MF–NF, and 99.7% for MF softening [87]. MF removes little due to limited adsorption and size exclusion. MF–NF adds charge-based rejection, while softening induces chemical precipitation, enhancing retention.

### 4.2. Limits and Potential of UF for Phosphorus Fractionation Removal

UF represents a highly versatile and advanced method for the removal of contaminants from liquid waste, including phosphorus compounds [96]. The fundamental principle underlying this method is size exclusion, whereby the membrane’s pore size plays the role of a physical barrier. This obstruction facilitates the selective permeation of water and smaller molecules while retaining larger phosphorus-containing particles [97]. This approach is particularly effective for particulate phosphorus and larger colloidal forms. The material composition and surface characteristics of the membrane are of great importance in this regard [98]. Membranes with charged or hydrophilic surfaces have the potential to enhance adsorption efficiency by attracting oppositely charged phosphate ions or other phosphorus species (Table 5). Due to its smaller pore size, UF offers better removal of phosphorus compounds (50–80%) [91]. When combined with coagulation, removal efficiencies of 90–99% can be achieved [99]. UF effectively separates phosphorus compounds by size exclusion mechanisms without the need for additional chemicals, thus minimizing secondary pollution concerns [100].

### 4.3. Phosphorus Speciation as the Determining Factor in MF and UF Performance

Table 4 and Table 5 reveal that phosphorus removal by MF and UF is strongly dependent on its association with organic or particulate matter. High TP retention is consistently observed when phosphorus is bound to solids or colloids, whereas dissolved orthophosphate largely permeates both membrane types. UF shows improved TP retention compared to MF, not due to direct phosphate rejection, but due to enhanced retention of phosphorus associated organic fractions. These findings confirm that membrane processes act as indirect phosphorus separators, emphasizing the need for upstream speciation control to achieve meaningful phosphorus recovery.

## 5. Nitrogen Removal from Aqueous Waste Streams

### 5.1. Microfiltration Constraints in the Separation of Nitrogen Species

Nitrogen compounds are ubiquitous in agricultural runoff, wastewater treatment plants, industrial effluents, and household waste, posing significant environmental challenges [105,106]. These compounds primarily include ammonia (NH_3_), nitrates (NO_3_^−^), nitrites (NO_2_^−^), and total Kjeldahl nitrogen (TKN) [19,107]. The separation of them from liquid waste using MF membranes is generally less effective when these membranes are used alone [108,109]. Nevertheless, when used in combination with other treatment processes, MF can contribute to nitrogen removal efficiencies [108,110]. The performances of MF for nitrogen compound removal are given in Table 6.

### 5.2. Form-Dependent Retention of Nitrogen During Ultrafiltration

The efficiency of UF in separating nitrogen compounds from liquid waste relays on several factors including membrane material and structure [111,112] (Table 7). Membrane materials can be organic polymers or inorganic substances tailored for specific separation needs [111]. Proper selection ensures optimal interaction between nitrogenous compounds and the membrane surface [112]. UF can contribute to nitrogen removal efficiencies of 80–95% when used in combination with other treatment processes [113]. Using UF technology, the liquid fraction of digestate pre-treated by electrocoagulation with Fe electrodes rejects 82% of NH_4_^+^ and 49% when using Al electrodes [114]. In their experimental work, ref. [115] found that the nitrogen efficiency of anaerobic digestate in the agricultural sector produced by pressure-driven UF is around 75–95% and 85–99%.

### 5.3. Intrinsic Limits of MF and UF for Dissolved Nitrogen Species

The data summarized in Table 6 and Table 7 demonstrate the intrinsic limitation of MF and UF membranes for removing dissolved nitrogen species such as ammonium and nitrate. Reported nitrogen removals are largely attributable to particulate or organically bound nitrogen rather than true rejection of ionic forms. UF exhibits slightly higher TN retention, primarily due to its ability to retain nitrogen-containing macromolecules. These observations confirm that MF and UF, when applied as standalone processes, are insufficient for comprehensive nitrogen separation and must be coupled with biological or physicochemical processes to transform dissolved nitrogen into membrane-retainable forms.

## 6. Cross-Compound Synthesis of MF and UF Fractionation Behavior

Beyond compound-specific performance, the comparative behavior of MF and UF across organic carbon, phosphorus, and nitrogen reveals consistent fractionation patterns governed by solute association, charge interactions, and secondary fouling layers. This section synthesizes these patterns to clarify when MF or UF provides functional advantages within integrated treatment systems. When evaluated through a mechanistic lens, MF and UF exhibit fundamentally different fractionation roles rather than a simple hierarchy of removal efficiency [57,116] (Table 8). MF primarily relies on size exclusion to retain suspended solids, particulate organic matter, and microorganisms, making it suitable for bulk solid–liquid separation [11]. However, its capacity to remove dissolved or low-molecular-weight compounds is limited. In contrast, UF membranes offer a tighter structure, which enables them to retain macromolecules, colloids, and certain dissolved organic and nutrient compounds through a combination of size exclusion, adsorption, and membrane–solute interactions [109]. The effectiveness of each technology depends on several factors including influent characteristics, membrane material and surface charge, transmembrane pressure, and pretreatment steps. For instance, UF generally outperforms MF in removing total phosphorus when a large fraction is in colloidal or organic bound forms, whereas MF shows comparable performance for particulate dominated streams. Moreover, UF membranes may exhibit better retention of nitrogen in the form of particulate nitrogen or colloid-bound ammonium, while both membranes are less effective for ionic species such as nitrate and phosphate unless supported by pretreatment or hybrid systems [68].

## 7. Conclusions

This review critically examined the performance of microfiltration and ultrafiltration membranes for the separation of organic carbon and nutrient compounds from liquid waste streams. The analysis confirmed the proposed hypothesis that the separation efficiency of MF and UF is not solely dictated by nominal pore size, but by the interplay between membrane characteristics, solute properties, feed composition, and operational conditions. MF membranes demonstrate robust performance in retaining particulate organic carbon and particulate-bound phosphorus and nitrogen, while UF membranes provide enhanced removal of macromolecular organic carbon, and colloidal or organically bound phosphorus. However, dissolved inorganic nitrogen and phosphorus species largely permeate both membrane types when applied as standalone processes, highlighting the intrinsic limitations related to charge effects, ionic speciation, and pore wetting phenomena. Importantly, this review shows that integrating MF and UF with pretreatment or post-treatment processes such as coagulation, adsorption, biological treatment, or chemical precipitation fundamentally alters the separation mechanisms and significantly enhances nutrient retention. In this context, membrane fouling, often regarded as a drawback, can act as a secondary selective layer that improves nutrient fractionation when properly controlled.

From a broader perspective, MF and UF should no longer be viewed solely as polishing or solid–liquid separation steps, but as strategic components of resource-oriented wastewater treatment systems. Their appropriate selection and integration enable the concentration of organic carbon and nutrients into recoverable streams, supporting circular economy objectives and decentralized treatment solutions.

Future research should focus on tailoring membrane surface properties, optimizing hybrid system configurations, and developing process design guidelines that explicitly target selective nutrient recovery rather than conventional removal, thereby advancing membrane-based technologies toward sustainable and resilient wastewater management. This reframing supports a shift from removal-oriented design toward selective fractionation strategies in membrane-based wastewater treatment.

## Figures and Tables

**Table 1:** Size of nutrients from wastewater [40,41].

Component	Nutrients	Nutrients Form	Size (µm)
Organics	Organic Carbon	TOC	1–100 μm
Inorganics	Nitrogen	TN	>0.5 nm
		Ammonium ion (NH_4_^+^)	0.1 to 0.5 nm
		Nitrate (NO_3_^−^)	0.2 to 0.4 nm
		Nitrite (NO_2_^−^)	0.2 to 0.4 nm
	Phosphorus	TP	higher than 0.5 nm
		Phosphate (PO_4_^3−^)	0.5 nm in diameter

**Table 2:** Applications of microfiltration for organic carbon fractionation in liquid waste.

Wastewater Type	Feed Characteristics	Membrane Properties	Operating Conditions	Removal Rate	References
Secondary treated water	pH: 6.8–7.5, Temp: 15–30 °C, Turbidity: 6–14 NTU, TSS: 8–12 mg/L, DOC: 6.3–8.4 mg/L	Plate, Polyolefin, 63 m^2^, 98 LMH, 50 × 100 × 0.5 cm, 0.22 µm	Suction/Rest: 8/2 min, Air flow: 10 LPM/module, Recovery: 98%, 30 cmHg, 30 LMH, Duration: 59 days	25–30% in DOC	[56]
Olive oil mill	COD: 120,000 mg/L, SS: 18,600 mg/L, Oil & Grease: 2870 mg/L, TOC: 46,340 mg/L	Cell body and cell holder	pH: 2–7, Pressure: 0–2 bar, Flow: 100–200 L/h, Time: 120 min	75.4% in TOC	[57]
Oil	TSS: 92 mg/L, Oil & Grease: 26 mg/L, TOC: 141 mg/L, Turbidity: 21 NTU	Ceramic membrane, Øext: 30 mm, Øchannel: 4 mm, Thickness: 1.5–2 mm, Surface: 0.24 m^2^, Pore: 0.2 µm	Max Temp: 1200 °C, Max Pressure: 10 bar, pH range: 0–14, Flux: >500 L/h·m^2^, Time: 90 min	higher than 95% in TOC	[58]
Oilfield	TOC: 386 mg/L, Naphthalene: 0.106 mg/L, Phenols: 4.3 mg/L	Mixed cellulose ester (MCE)	Ambient Temp, Feed Velocity: 0.43 m/s, Pressure: 50 kPa, Time: 210 days	82% in TOC	[59]
Industrial textile	TOC: 690.3 mg/L, COD: 1213.9 mg/L, BOD: 975.4 mg/L	Phosphate/Kaolinite membrane, Porosity: 41.3%, Pore: 0.35 µm	Sintering Temp: 900–1000 °C, Pressure: 40.2 MPa, Permeability: 1045 L/h·m^2^·bar, Time: 2–4 h	69.39% in TOC	[60]
Oily	TOC: 500 mg/L	Ceramic (Al_2_O_3_), Height: 425 mm, Diameter: 30 mm, 19 lumens Pore: 50 nm, Thickness: 20–50 nm, Area: 0.1 m^2^	CFV: 1.68 m/s, pH increase: 3.8–5.8, Pressure: 0.05–0.30 MPa, Flux: 163–141 L/m^2^ h	96.6–97.7% in TOC	[61]
Domestic	COD: 122 mg/L, BOD5: 59 mg/L, TOC: 4 mg/L, SS: 91 mg/L, Turbidity: 0.15 NTU, Color: 42 CU	Effective Area: 4 m^2^, Porosity: 71%, Dimensions: 788 × 486 × 48 mm, Pore: 0.1 µm	Max capacity: 8 m^3^/d, Suction: 10 min, Idle: 2 min (variable), Recovery: 98%, Agitator speed: 350 rpm (variable), Rotation: 8 s, Pressure: 20–50 kPa, Initial flux: 0.02 L/m^2^·h, Time: 120 days	65.8% in TOC and 60% in DOC	[62]
Reclamation/reuse	pH: 6.8–7.5, Temp: 15–30 °C, Turbidity: 6–14 NTU, TSS: 8–12 mg/L, COD: 6.3–8.4 mg/L	MF Plate, Polyolefin, Pore size: 0.22 µm, Area: 63 m^2^, flux: 98 LMH, Size: 50 × 100 × 0.5 cm	Tank dimensions: 1600 × 2200 × 3000 mm, Mode: Constant flow filtration, Flux: 30 LMH, Suction/Rest: 8/2 min	25–30%; 20–25% of COD	[56]
Reclamation/reuse	pH: 6.8–7.5, Temp: 15–30 °C, Turbidity: 6–14 NTU, TSS: 8–12 mg/L, COD: 6.3–8.4 mg/L	MF + GAC, Area: 1100 m^2^, Density: 440 kg/m^3^, Pore size: 0.60 µm	Tank: 1600 × 2200 × 3000 mm, Mode: Constant flow, Flux: 30 LMH, Suction/Rest: 8/2 min, Air flow: 10 LPM/module, Recovery: 99%, Duration: 130 days	53% of COD	[56]
Secondary effluent	pH: 6–8, COD: 23–43 mg/L, TP: 2.35–2.54 mg/L, NKT: 1.08–1.14 mg/L	10-inch module, Polypropylene fibers, Pore size: 1 µm	Rapid mixing: 30 s, Slow mixing: 10 min, Settling: 20 min, Total time: 40 min	78% of COD	[57]
Activated sludge	TCOD: 223.6 mg/L, NH_4_^+^: 28.3 mg/L, NO_3_^−^: 0.2 mg/L, PO_4_^3−^: 2.0 mg/L	Flat-sheet MF membrane, Pore size: 0.2 µm, Material: Polyethersulfone, Hydrophilic	Qin: 72 m^3^/day, Recycling flow (Or): 195 m^3^/day (2.7 × Qin), Flux: 15.4 L/m^2^·h, SRT: 80 days, Anoxic/Anaerobic ratio: 4:2 h, Duration: 80 days	96.3% of TCOD	[63]
Poultry Slaughterhouse	TSS: 2016 mg/L, TDS: 2012 mg/L, COD: 34 mg/L	pH range: 1–11, Pore size: 0.2 µm, PVDF, Dimensions: 0.127 × 0.1 × 0.083 m, Area: 0.0042 m^2^	Pressure: 413 kPa, Flow rate: 6.0 L/min	26.5% of COD	[16]

**Table 3:** Ultrafiltration-based approaches for organic carbon separation in liquid waste.

Wastewater Type	Feed Characteristics	Membrane Properties	Operating Conditions	Removal Rate	References
Oil and grease	TOC: 81 mg/L, TSS: 60 mg/L, COD: 124 mg/L, Turbidity: 53 NTU, TDS: 420 mg/L	Length: 12 cm, Diameter: 0.8–1.1 mm, Thickness: 100–150 mm, Area: 35.8 cm^2^, Pore size: 1.10 µm	Temperature: 25 °C, CFV: 1 m/s, Pressure: 3 bar, Flux: 84.1 L/m^2^·h, Duration: 8 h	96.3% in TOC	[71]
Oily	TSS: 60 mg/L, TDS: 2028 mg/L, Oil & Grease: 78 mg/L, COD: 124 mg/L, BOD_5_: 52 mg/L, TOC: 81 mg/L, Turbidity: 53 NTU	Membranes: Polysulfone (PS, 30 kDa), Polyacrylonitrile (PAN, 20 kDa), Pore size: 30 kDa	CFV: 1 m/s, Temp: 40 °C, pH: 9, TMP: 3 bar, Flux: 45 L/m^2^·h	99.7% in TOC	[73]
Municipal	pH: 7.44–7.88, COD: 39–57 mg/L, BOD_5_: 12–19 mg/L, SS: 14–17 mg/L, N–NH_4_^+^: 0.01–0.03 mg/L	Hydrophilicity: 4, pH range: 1.5–12, Max pressure: 30 bar, Temp: 65 °C, Solvent resistance: Medium, Pore size: 4.13 nm	Flow: 5–50 mL/min, Pressure: 1–4 bar, ΔP at 2 m/s: 15 kPa, ΔP at 4 m/s: 50 kPa, Permeability: 10.74 L/m^2^·h·bar, Time: 1–4 h	up to 50% in terms of COD and TOC	[74]
Oily from an oil field	COD: 637 mg/L, Oil: 15.5 mg/L, TOC: 214.9 mg/L, Turbidity: 98 NTU, SS: 15.8 mg/L	Tubular cross-flow membrane, filter area: 0.01256 m^2^, MWCO: 35 kDa, pore size: 0.45 μm	TMP: 0.1 MPa, Temp: 30 °C, Feed CFV: 7.8 m/s, Flux: 170.06 L/m^2^ h–149.81 L/m^2^ h	98% in TOC	[75]
Oily	COD: 52,700 mg/L, TOC: 13,800 mg/L	MWCO: 30,000 Daltons; Surface area: 2.2 m^2^	Temp: 40–50 °C, Flux: 53 L/m^2^·h	93.5% in TOC	[73]
Vegetable oil	COD: 550 ppm, TOC: 300 ppm, TSS: 110 ppm, PO_4_^3−^: 20 ppm, Cl: 350 ppm, pH: 10.5	MWCO: 30 kDa	Temp: 30 °C, Concentration: 300 ppm, Velocity: 0.33 m/s, pH: 10.5; Pressure: 2 bar; Flux: 50 L/m^2^·h; Time: 70 min	87% in TOC	[76]
Poultry Slaughterhouse	TSS: 2016 mg/L, SS: 4 mg/L, TDS: 2012 mg/L, COD: 34 mg/L	pH: 1–11, MWCO: 3000 Da, Polymer: PES, Dimensions (L × W × H): 0.127 m × 0.1 m × 0.083 m, Area: 0.0042 m^2^	Pressure: 1239 kPa; Flow rate: 6.0 L/min	8.8% of COD	[16]
Influent from the treatment plant	COD: 237 mg/L, BOD_5_: 78.4 mg/L, NH_4_^+^: 16.14 mg/L, TP: 2.71 mg/L, SS: 146 mg/L	Material: PVDF, Pore size: 0.1 µm, Area: 80 m^2^, Nominal size: Φ225 × 2360 mm, Resistance to NaClO: 5000 ppm	Permeability: 1.5 LMH/kPa	78% of COD and 91% of BOD_5_	[77]
Pig manure	TS: 20,000 mg/L, SS: 13,000 mg/L, TCOD: 30,000 mg/L, TP: 160 mg/L	Area: 0.1 m^2^, internal diameter: 2 cm, Material: PVDF, Pore size: 0.01 µm	Initial water flux: 600–1080 L/m^2^·h; Operation mode: batch with recirculation; pH: 6.0–8.0; Temp: 15–25 °C; Operation time: 24 h; Concentration factor: 2.1–3.7 L/L; TMP: 100–180 kPa; Tangential velocity: 2.9–3.3 m/s.	TCOD = 15,000 mg/L	[78]
Sieved and settled manure supernatant (SAS)	TS: 12,000 mg/L, SS: 7000 mg/L, TCOD: 41,000 mg/L, CBOD_2_: 16,000 mg/L	Area: 0.1 m^2^, Material: PVDF, pore size: 0.01 µm, internal diameter: 3 cm.	Mean operation time: 24 h; Concentration factor: 3.1–3.7; TMP: 100–175 kPa; Tangential velocity: 3.0–3.3 m/s.	TCOD = 20,000 mg/L	[78]
Sieved, biologically treated, and SBS	TS: 2500 mg/L, SS: 500 mg/L, TCOD: 500 mg/L, CBOD_2_: 130 mg/L	0.1 m^2^ of area, PVDF, MWCO: 100,000 Da, single tube with 4 cm internal diameter	Mean operation time: 10 h; Concentration factor: 4.3–9; TMP: 155–210 kPa; Tangential velocity: 2.6–3.5 m/s	Total COD = 160 mg/L	[78]
Biologically treated wastewater	pH: 7.8, EC: 400 µS/m, TSS: 300 mg/L, COD: 12 mg/L, BOD_5_: 4.5 mg/L	Material: zirconium oxide, Area: 50 cm^2^, Pore size: 50 nm, I.D.: 7 mm, Length: 25 cm	Forward filtration TMP: 1.8 bar, CFV: 7 m/s, Backflush duration: 0.5–1 s, Backflush intervals: 1–2 min, Temperature: 20 °C	52% of COD; 45% of BOD_5_	[79]
Vegetable oil	COD: 550 ppm, TOC: 300 ppm, TSS: 120 ppm, PO_4_^3−^: 20 ppm, Cl^−^: 350 ppm	Polymeric membrane made of polysulfone, MWCO: 30 kDa	Pressure difference: >3 bar, high cross flow velocity, temp: 30 °C, pH: 9	91% in COD; 87% in TOC	[80]
Urban	COD: 50–2234 mg/L, SS: 80–1327 mg/L, NH_3_-N: 10–40 mg/L, Turbidity: 50–80 NTU, pH: 7.5–8.5, Temp: 15–25 °C	Materials: ZrO_2_ and γ-Al_2_O_3_, diameter: 4.5 mm, length: 40 cm, Area: 0.04 m^2^, pore size: 0.02 µm, MWCO: 300,000 Da, initial permeability: 4–5 L/m^2^·h·kPa	162-day pilot-scale operation, sludge retention time (SRT): 5, 15, and 30 days, hydraulic retention time (HRT): 5 h, membrane flux: 75–150 L/m^2^·h	97% of COD	[81]
Anaerobically digested sludge	pH: 7.68, EC: 7.15 mS/cm, Turbidity: 166.15 NTU, TCOD: 988.5 mg/L, NH_4_^+^: 472.75 mg/L	Pore size: 0.01 μm flat sheet membrane, total active surface area 0.0125 m^2^	Fixed CFV: 2 m/s, TMP varied between 1 and 2 bar	66% TCOD removal	[82]
Raw sewage	EC: 1340 ± 197 µS/m, Turbidity: 110.02 ± 37.44 NTU, TSS: 63 ± 41 mg/L, COD: 218 ± 43 mg/L, PO_4_^3−^: 4.1 ± 1 mg/L	PVDF membranes, 5 tubes of 5.2 mm diameter, 1 m module length, Area: 0.073 m^2^	TMP values: 0.3, 0.5, 1.0 bar, CFV: 2.0 m/s, time: 30 min	138 ± 26 mg/L of COD	[83]
Primary clarifier effluent	EC: 1042 ± 316 µS/m, Turbidity: 54.62 ± 24.58 NTU, TSS: 46 ± 18 mg/L, COD: 135 ± 37 mg/L, NH_4_^+^: 29.9 ± 9.8 mg/L, PO_4_^3−^: 3.6 ± 2.4 mg/L	PVDF membranes, 5 tubes of 5.2 mm diameter, 1 m module length, total filtration surface area: 0.073 m^2^	TMP values: 0.3, 0.5, 1.0 bar, CFV: 2.0 m/s, time: 30 min	78 ± 30 mg/L of COD	[83]
Urban	pH: 8.3, Temp: 22.9 °C, EC: 1486 µS/m, TSS: 21 mg/L, Turbidity: 73 NTU, COD: 80 mg/L, BOD: 61 mg/L, NH_4_^+^: 18 mg/L, NO_2_-N: 0.10 mg/L, NO_3_^−^: 9 mg/L, PO_4_^3−^: 4.2 mg/L	Pore size: 50,000 MWCO, Membrane: Hydrophilic, Material: Polyolefin, Area: 23.2 m^2^, TMP: 200 kPa	Permeate flow below 0.2 m^3^/h, experiment periods: 115 h at 260 kPa, 395 h at 650 kPa, 83 h at 520 kPa, safety stops filtration above 700 kPa or 40 °C	43 mg/L 0 f COD and 17 mg/L of BOD_5_	[84]

**Table 4:** Microfiltration pathways for phosphorus-containing fractions in liquid waste.

Wastewater	Feed Characteristics	Membrane Properties	Operating Conditions	Removal Rate	References
Secondary treated water	pH: 6.8–7.5, Temp: 15–30 °C, Turbidity: 6–14 NTU, TSS: 8–12 mg/L, UV260: 0.25–0.35 1/cm, DOC: 6.3–8.4 mg/L	Material: Polyolefin, Area: 63 m^2^, Flux: 98 LMH, Composition: 50 × 100 × 0.5 cm, Pore size: 0.22 µm	Suction/rest: 8/2 min, Air flow: 10 LPM/module, Recovery: 98%, Vacuum: 30 cmHg, Flux: 30 LMH, Duration: 59 days	5–8% of TP	[56]
Reclamation/reuse	pH: 6.8–7.5, Temp: 15–30 °C, Turbidity: 6–14 NTU, TSS: 8–12 mg/L, UV260: 0.25–0.35 1/cm, DOC: 6.3–8.4 mg/L	Pore size: 0.22 µm, Material: Polyolefin, Area: 63 m^2^, Flux: 98 LMH, Composition (L × H × T): 50 × 100 × 0.5 cm	Submerged cylindrical tank (1600 × 2200 × 3000 mm), constant flow filtration, flux: 30 LMH, suction/rest: 8/2 min, air flow: 10 LPM/module, recovery: 98%, duration: 70 days	5–8% of TP	[56]
Reclamation/reuse	pH: 6.8–7.5, Temp: 15–30 °C, Turbidity: 6–14 NTU, TSS: 8–12 mg/L, UV260: 0.25–0.35 1/cm, DOC: 6.3–8.4 mg/L	Area: 1100 m^2^, density: 440 kg/m^3^, Pore size: 0.60 µm, uniformity coefficient: 1.9, particle size: >12 mesh (1.70 mm)	Submerged cylindrical tank: 1600 × 2200 × 3000 mm, operation mode: constant flow filtration, flux: 30 LMH, suction/rest: 8/2 min, air flow: 10 LPM/module, recovery: 99%, duration: 130 days	13% of TP	[56]
Sedimentation pond	pH: 6–8, DOC: 23–43 mg/L, SST: 8–10 mg/L, PT: 2.35–2.54 mg/L, NKT: 1.08–1.14 mg/L	10-inch module with polypropylene fibers, pore size: 1 µm	Rapid mixing: 30 s, low mixing: 10 min, settling: 20 min; total time: 40 min	7% of TP	[57]
Activated sludge floc	TCOD: 223.6 mg/L, TN: 31.5 mg/L, NH_4_^+^: 28.3 mg/L, NO_3_^−^: 0.2 mg/L, TP: 3.0 mg/L, PO_4_^3−^: 2.0 mg/L	Flat-sheet microfiltration membrane, pore size: 0.2 µm, made of hydrophilic polyethersulfone	HRT: 2.9 h (SAAR), 3.5 h (MBR), Internal recycling flowrate: 195 m^3^/day (2.7 × Qin during anoxic time), Flux: 15.4 L/m^2^·h, Influent flowrate (Qin): 72 m^3^/day, SRT: 80 days, Recycling time anoxic to anaerobic ratio: 4:2	82.6% of TP and 70.8% of PO_4_^3−^	[63]
From Automobile plant	PO_4_^3−^: 100–118 mg/L, Zn^2+^: 1.26 mg/L, Ca^2+^: 134.88 mg/L, Mg^2+^: 15.95 mg/L, Alkalinity as CaCO_3_: 120 mg/L, TDS: 1325 mg/L, SS: 250 mg/L, pH: 6.8	Al_2_O_3_ ceramic membrane, Thickness: 20 μm, nominal pore size: 0.2 μm, Area: 0.05 m^2^	Transmembrane pressure: 0.15 MPa, cross flow velocity: 2.1 m/s, time: 2 h	99.7% of PO_4_^3−^	[93]
Phosphoric acid	Phosphoric acid, 85% concentration in water	Carbon membranes, thickness: 0.3 cm, diameter: 2.5 cm	Drying in ambient conditions, pyrolysis at 650 °C for 1 h, total time: 240 min	55.3% in acid form	[94]
Liquid crystal display	pH: 4.72, EC: 424 µS/cm, TSS: 0.58 mg/L, Turbidity: 14.4 NTU, PO_4_^3−^: 187.6 mg/L	Mixed cellulose ester hydrophilic (MCE) membranes with pore sizes of 0.22 µm and 0.45 µm	Precipitation MF, Temp: 25 °C, Pressure: 0.5 and 1.0 bar, CFV: 0.48 m/s (laminar flow, Re = 1618) and 0.96 m/s (turbulent flow, Re = 3234), Time: 100 min	99% of PO_4_^3−^	[95]
Poultry Slaughterhouse	TSs: 2016 mg/L, TDSs: 2012 mg/L, COD: 34 mg/L, TP: 54 mg/L, TN: 252 mg/L	Pore size: 0.2 µm, Polymer: PVDF, Dimensions: 0.127 m (L) × 0.1 m (W) × 0.083 m (H), Area: 0.0042 m^2^	Pressure: 413 kPa, Flow rate: 6.0 L/min (MF membrane)	5.6% of TP	[16]
Urban wastewater tertiary	pH: 7.9, Temp: 16.6 °C, EC: 1748 µS/m, TSS: 16 mg/L, Turbidity: 51 NTU, NH_4_^+^: 29 mg/L, NO_2_^−^: 0.20 mg/L, NO_3_^−^: 0.50 mg/L, TP: 8.8 mg/L, PO_4_^3−^: 6.4 mg/L	Pore size: 0.2 µm, Material: Propylene, Area: 30 m^2^, TMP: 100 kPa	Continuous operation with steady flow (1.6 m^3^/h) and 22 min filtration cycles, Total runtime: 1000 h, TMP around 40 kPa with pressure spikes before cleaning	7.6 mg/L in TP and 5.9 mg/L of PO_4_^3−^	[84]

**Table 5:** Ultrafiltration behavior toward phosphorus compounds in liquid waste.

Wastewater Type	Feed Characteristics	Membrane Properties	Operating Conditions	Removal Rate	References
Car wash		Zirconia oxide	Pressure: 250 kPa, Flux: 100 L/m^2^ h, coagulant: FeCl_3_	Phosphorus removal: 100%	[101]
Aqueous solution	Water content, % = 69.7	Porosity: 42.8%, Mean pore size: 9.13 nm	pH: 2.0, Pressure: 2 bar, Operation: Dead-end ultrafiltration	93.6% of PO_4_^3−^	[102]
Municipal	TP: 1.11 mg/L, BOD: 9.9 mg/L	Pore size: 0.02–0.04 μm, hydraulic loading rate: 7.52 L/sm^2^, flow rate capacity: 5.68 × 10^5^ L/d		>96% in TP	[103]
Poultry Slaughterhouse	TSs: 2016 mg/L, TSSs: 4 mg/L, TDSs: 2012 mg/L, COD: 34 mg/L, TP: 54 mg/L, TN: 252 mg/L	Pore size: 30,000 Da, Polymer: PES, Dimensions: 0.127 × 0.1 × 0.083 m, Area: 0.0042 m^2^	Pressure: 1239 kPa, Flow rate: 6.0 L/min	16.7% in TP	[16]
Forms micelles		Hollow fiber acrylonitrile membrane, surface area: 0.088 m^2^, MWCO: 30,000 Da and 10,000 Da	25 °C, stirred at 100 rpm, TMP: 2 bar, duration: 25 h	>91% of PO_4_^3−^	[104]
From the treatment plant	BOD_5_: 78.4 mg/L, NH_4_^+^: 16.14 mg/L, TP: 2.71 mg/L, SS: 146 mg/L	Material: PVDF, Pore size: 0.1 µm, Area: 80 m^2^, Nominal size: Φ225 × 2360 mm, Resistance to NaClO: 5000 ppm	Permeability: 1.5 LMH/kPa	85% in TP	[77]
Pig manure	TS: 20,000 mg/L, SS: 13,000 mg/L, TCOD: 30,000 mg/L, TP: 160 mg/L	Area: 0.1 m^2^, Material: PVDF, Pore size: 0.01 µm, single tube, internal diameter: 2 cm	Initial flux: 600–1080 L/m^2^·h, pH: 6.0–8.0, Temp: 15–25 °C, Mean operation time: 24 h, Concentration factor range: 2.1–3.7; TMP: 100–180 kPa, CFV: 2.9–3.3 m/s	TP = 80 mg/L	[78]
Sieved and settled manure	TS: 12,000 mg/L, SS: 7000 mg/L, TCOD: 41,000 mg/L, TKN: 1800 mg/L, TP: 1190 mg/L	Material: PVDF, Pore size: 0.01 m, single tube, internal diameter: 3 cm	Mean operation time: 24 h, Concentration factor range: 3.1–3.7 L/L, TMP: 100–175 kPa, CFV: 3.0–3.3 m/s	TP = 150 mg/L	[78]
Sieved and biologically treated	TS: 2500 mg/L, SS: 500 mg/L, TCOD: 500 mg/L, TKN: 695 mg/L, TP: 50.5 mg/L	Material: PVDF, Pore size: 0.01 m, single tube, internal diameter: 4 cm	Mean operation time: 10 h, Concentration factor range: 4.3–9; TMP: 155–210 kPa, CFV: 2.6–3.5 m/s	TP = 30 mg/L	[78]
Biologically treated	pH: 7.8, EC: 400 µS/m, TDS: 300 mg/L, COD: 12 mg/L, BOD_5_: 4.5 mg/L, TP: 1.2 mg/L, TN: 4 mg/L	Material: zirconium oxide, Area: 50 cm^2^, Pore size: 50 nm, I.D.: 7 mm, Length (L): 25 cm	Forward filtration TMP: 1.8 bar, CFV: 7 m/s, Backflush duration: 0.5 or 1 s, Backflush intervals: 1 or 2 min, Temp: 20 °C	25% of TP	[79]
Biologically treated	pH: 7.8, EC: 400 µS/m, TDS: 300 mg/L, COD: 12 mg/L, BOD_5_: 4.5 mg/L, TP: 1.2 mg/L, TN: 4 mg/L	Material: zirconium oxide, Area: 50 cm^2^, Pore size: 50 nm, I.D.: 7 mm, Length (L): 25 cm	Forward filtration TMP: 1.8 bar, CFV: 7 m/s, Backflush duration: 0.5–1 s, Backflush intervals: 1–2 min, Temp: 20 °C	55% of TP	[79]
Vegetable oil	COD: 550 ppm, TOC: 300 ppm, TSS: 110 ppm, PO_4_^3−^: 20 ppm, Cl^−^: 350 ppm, pH: 10.5	Material: Polymeric (Polysulfone), MWCO: 30 kDa	Pressure difference: >3 bar, CFV, Temp: 30 °C, pH: 9	85% of PO_4_^3−^	[76]
Municipal: raw sewage	EC: 1340 ± 197 µS/m, Turbidity: 110.02 ± 37.44 NTU, TSS: 63 ± 41 mg/L, COD: 218 ± 43 mg/L, TP: 5.4 ± 0.9 mg/L, TN: 32.3 ± 5.2 mg/L, NH_4_^+^: 38.4 ± 12.9 mg/L, PO_4_^3−^: 4.1 ± 1 mg/L	Material: PVDF, diameter: 5.2 mm, 5 tubes, 1 m module, total filtration surface: 0.073 m^2^	TMP: 0.3, 0.5, 1.0 bar, CFV: 2.0 m/s, Time: 30 min	4.4 ± 0.6 mg/L in TP and 4 ± 0.8 mg/L of PO_4_^3−^	[83]
Municipal: primary clarifier effluent	EC: 1042 ± 316 µS/m, Turbidity: 54.62 ± 24.58 NTU, TSS: 46 ± 18 mg/L, COD: 135 ± 37 mg/L, TP: 5 ± 0.8 mg/L, TN: 23.8 ± 2.3 mg/L, NH_4_^+^: 29.9 ± 9.8 mg/L, PO_4_^3−^: 3.6 ± 2.4 mg/L	Material: PVDF, diameter: 5.2 mm, 5 tubes, 1 m module length, total filtration surface: 0.073 m^2^	TMP values: 0.3, 0.5, and 1.0 bar; CFV: 2.0 m/s; operation time: 30 min	4.1 ± 1 mg/L in TP and 3.4 ± 1.6 mg/L of PO_4_^3−^	[83]
Urban	pH: 8.3, Temp: 22.9 °C, EC: 1486 µS/cm, TSS: 21 mg/L, Turbidity: 73 NTU, COD: 80 mg/L, BOD: 61 mg/L, TN: 43 mg/L, NH_4_^+^: 18 mg/L, NO_2_^−^: 0.10 mg/L, NO_3_^−^: 9 mg/L, PO_4_^3−^: 4.2 mg/L	MWCO: 50 kDa, Material: polyolefin, Area: 23.2 m^2^, Mode: cross-flow, Pressure: 200 kPa	Permeate flow: 0.2 m^3^/h, pressure: 700 kPa	4 mg/L of PO_4_^3−^	[84]

**Table 6:** Microfiltration response to nitrogen species in liquid waste.

Wastewater	Feed Characteristics	Membrane Properties	Operating Conditions	Removal Rate	References
Secondary treated	pH: 6.8–7.5, Temp: 15–30 °C, Turbidity: 6–14 NTU, TSS: 8–12 mg/L, UV260: 0.25–0.35 1/cm, DOC: 6.3–8.4 mg/L	Material: Polyolefin, Area: 63 m^2^, Dimensions: 50 × 100 × 0.5 cm, Pore size: 0.22 µm	Suction/rest: 8/2 min, Air flow: 10 LPM/module, Recovery: 98%, Vacuum: 30 cmHg, Flux: 30 LMH, Duration: 59 days	5–10% of TN	[56]
Reclamation/reuse	pH: 6.8–7.5, Temp: 15–30 °C, Turbidity: 6–14 NTU, TSS: 8–12 mg/L, UV260: 0.25–0.35 1/cm, DOC: 6.3–8.4 mg/L	Material: Polyolefin, Pore size: 0.22 µm, Area: 63 m^2^, Flux: 98 LMH, Dimensions: 50 × 100 × 0.5 cm	Tank dimensions: 1600 × 2200 × 3000 mm, Flux: 30 LMH, Suction/rest cycle: 8/2 min, Air flow: 10 LPM/module, Recovery: 98%, Operation duration: 70 days	5–10% of TN	[56]
Secondary effluent discharged	pH: 6–8, DOC: 23–43 mg/L, SST: 8–10 mg/L, TP: 2.35–2.54 mg/L, NKT: 1.08–1.14 mg/L	10-inch module with polypropylene fibers, pore size of 1 µm	Rapid mixing for 30 s, followed by low mixing for 10 min, then settling for 20 min, totaling 40 min	40% of TKN	[57]
Activated sludge floc	TCOD: 223.6 mg/L, TN: 31.5 mg/L, NH_4_^+^: 28.3 mg/L, NO_3_^−^: 0.2 mg/L, TP: 3.0 mg/L, PO_4_^3−^: 2.0 mg/L	Pore size: 0.2 µm, material: polyethersulfone, hydrophilic	Flowrate: 195 m^3^/day, Flux: 15.4 L/m^2^·h, Influent flowrate: 72 m^3^/day, SRT: 80 days, Recycling time ratio: Anoxic: Anaerobic 4:2 h, Duration: 80 days	68.1% of TN; 95.3% in NH_4_^+^ and 9.7% of NO_3_^−^	[63]
Urban	pH: 7.9, Temp: 16.6 °C, EC: 1748 µS/m, TSS: 16 mg/L, Turbidity: 51 NTU, COD: 51 mg/L, BOD: 47 mg/L, TN: 34 mg/L, NH_4_^+^: 29 mg/L, NO_2_^−^: 0.20 mg/L, NO_3_^−^: 0.50 mg/L, TP: 8.8 mg/L, PO_4_^3−^: 6.4 mg/L	Type of membrane: Hollow fiber, Pore size: 0.2 µm, Material: propylene, Area: 30 m^2^, TMP: 100 kPa	Continuous operation, steady input flow: 1.6 m^3^/h, filtration time: 22 min, retro-cleansing and soaking: 1.9 min, total duration: ~1000 h, TMP: 40 kPa with peaks before CIP	35 mg/L of TN; 25 mg/L in NH_4_^+^; 3.2 mg/L of NO_3_^−^; 1.1 mg/L of NO_2_^−^	[84]

**Table 7:** Ultrafiltration response to nitrogen species in liquid waste.

Wastewater	Feed Characteristics	Membrane Properties	Operating Conditions	Removal Rate	References
Poultry Slaughterhouse	TSS: 2016 mg/L and 4 mg/L, TDS: 2012 mg/L, COD: 34 mg/L, TP: 54 mg/L, TN: 252 mg/L	Pore size: 0.2 µm, Material: PVDF, Dimensions: 0.127 × 0.1 × 0.083 m, Area: 0.0042 m^2^	Pressure: 413 kPa, Flow rate: 6.0 L/min	32.1% of TN	[16]
Forms micelles		Hollow fiber acrylonitrile membrane, Area: 0.088 m^2^, MWCO: 30,000 Da	Temp: 25 °C, Stirring: 100 rpm, TMP: 2 bar, Duration: 25 h	>86% of NH_4_^+^	[104]
Influent from the treatment plant	BOD_5_: 78.4 mg/L, NH_4_^+^: 16.14 mg/L, TP: 2.71 mg/L, SS: 146 mg/L	Material: PVDF, Pore size: 0.1 µm, Area: 80 m^2^, Nominal size: Φ225 × 2360 mm, Resistance to NaClO: 5000 ppm	Permeability: 1.5 LMH/kPa	98% of NH_4_^+^	[77]
Sieved and settled manure supernatant (SAS)	TS: 12,000 mg/L, SS: 7000 mg/L, TCOD: 41,000 mg/L, CBOD: 16,000 mg/L, TKN: 1800 mg/L, TP: 1190 mg/L	Material: PVDF, Pore size: 0.01 µm, single tube, I.D.: 3 cm	Mean operation time: 24 h, Concentration factor range: 3.1–3.7; TMP: 100–175 kPa, CFV: 3.0–3.3 m/s	TKN = 900 mg/L	[78]
Biologically treated	pH: 7.8, EC: 400 µS/m, TDS: 300 mg/L, COD: 12 mg/L, BOD_5_: 4.5 mg/L, TP: 1.2 mg/L, TN: 4 mg/L	Material: zirconium oxide, Area: 50 cm^2^, Nominal pore size: 50 nm, I.D.: 7 mm, Length: 25 cm	Forward filtration, TMP: 1.8 bar, CFV: 7 m/s, Temperature: 20 °C	10% of TN	[79]
Urban	COD: 50–2234 mg/L, SS: 80–1327 mg/L, NH_4_^+^: 10–40 mg/L, Turbidity: 50–80 NTU, pH: 7.5–8.5, Temp: 15–25 °C	Material: zirconia (ZrO_2_), diameter: 4.5 mm, length: 40 cm, Area: 0.04 m^2^, pore size: 0.02 µm, Permeability: 4–5 L/m^2^·h·kPa	162-day pilot-scale operation, SRT: 5, 15, 30 days, HRT: 5 h, Membrane flux: 75–150 L/m^2^·h	96.2% of NH_4_^+^	[81]
Anaerobically digested sludge	pH: 7.68, EC: 7.15 mS/cm, Turbidity: 166.15 NTU, TCOD: 988.5 mg/L, TN: 517.0 mg/L, N soluble: 429.50 mg/L, NH_4_^+^: 472.75 mg/L	Material: PES and PVDF, Area: 0.0125 m^2^	Fixed CFV: 2 m/s, TMP varied between 1 and 2 bar	13% of NH_4_^+^	[82]
Municipal: raw sewage ween	EC: 1340 µS/m, Turbidity: 110.02 NTU, TSS: 63 mg/L, COD: 218 mg/L, TP: 5.4 mg/L, TN: 32.3 mg/L, NH4^+^: 38.4 mg/L, PO_4_^3−^: 4.1 mg/L	Material: PVDF, diameter: 5.2 mm, 5 tubes, total filtration surface: 0.073 m^2^	TMP: 0.3, 0.5, 1.0 bar; CFV: 2.0 m/s; time: 30 min	29 ± 3 mg/L in TN and 39.4 ± 11.6 mg/L of NH_4_^+^	[83]
Municipal of primary clarifier effluent	EC: 1042 ± 316 µS/m, Turbidity: 54.62 ± 24.58 NTU, TSS: 46 ± 18 mg/L, COD: 135 ± 37 mg/L, TP: 5 ± 0.8 mg/L, TN: 23.8 ± 2.3 mg/L, NH_4_^+^: 29.9 ± 9.8 mg/L, PO_4_^3−^: 3.6 ± 2.4 mg/L	Material: PVDF, diameter: 5.2 mm, 5 tubes, 1 m module length, total filtration surface: 0.073 m^2^	TMP values: 0.3, 0.5 and 1.0 bar, CFV: 2.0 m/s, operation time: 30 min	28.1 ± 2.3 mg/L of TN	[83]
Urban tertiary	pH: 8.3, Temperature: 22.9 °C, EC: 1486 µS/cm, TSS: 21 mg/L, Turbidity: 73 NTU, COD: 80 mg/L, BOD: 61 mg/L, TN: 43 mg/L, NH_4_^+^: 18 mg/L, NO_2_^−^: 0.10 mg/L, NO_3_^−^: 9 mg/L, TP: 9.5 mg/L, PO_4_^3−^: 4.2 mg/L	Pore size: 50,000 MWCO, Hydrophilic, Material: Polyolefin, Area: 23.2 m^2^, TMP: 200 kPa	Permeate flow: 0.2 m^3^/h, Temp rose above 40 °C.	38 mg/L of TN; 19 mg/L in NH_4_^+^; 12 mg/L of NO_3_^−^ and 1.3 mg/L of NO_2_^−^	[84]

**Table 8:** Comparative performance of MF and UF membranes.

Membrane	Target Compounds	Removal Efficiency (%)	Main Mechanism	Advantages	Limitations
MF	TOC, TSS, particulate TP	60–75	Size exclusion	Low cost, simple operation, high flux	Limited dissolved nutrient removal
UF	TOC, TP, TN (particulate/colloidal)	75–99	Size exclusion + adsorption	High selectivity, broader retention range	Fouling sensitivity, higher cost

## Data Availability

No data created.

## Abbreviations

The following symbols and abbreviations are used in this manuscript:

BOD	Biochemical oxygen demand, 5 days
COD	Chemical oxygen demand
DOC	Dissolved organic carbon
MF	Microfiltration
OC	Organic carbon
PAC	Powdered activated carbon
PN	Particulate nitrogen
POC	Particulate organic carbon
PP	Particulate phosphorus
PVDF	Polyvinylidene fluoride
SS	Suspended solids
TCOD	Total chemical oxygen demand
TKN	Total Kjeldahl nitrogen
TMP	Transmembrane pressure
TN	Total nitrogen
TOC	Total organic carbon
TP	Total phosphorous
TSS	Total suspended solids
UF	Ultrafiltration
